# Body-Worn IMU Human Skeletal Pose Estimation Using a Factor Graph-Based Optimization Framework

**DOI:** 10.3390/s20236887

**Published:** 2020-12-02

**Authors:** Timothy McGrath, Leia Stirling

**Affiliations:** 1 Department of Aeronautics and Astronautics, Massachusetts Institute of Technology, 77 Massachusetts Avenue, Cambridge, MA 02139, USA; 2 Industrial and Operations Engineering, Robotics Institute, University of Michigan, 1205 Beal Avenue, Ann Arbor, MI 48109, USA; leias@umich.edu

**Keywords:** inertial measurement system, human motion, joint angle, self-calibrating, biomechanics, optimization, factor graph, knee, soft tissue artifacts

## Abstract

Traditionally, inertial measurement units- (IMU) based human joint angle estimation requires a priori knowledge about sensor alignment or specific calibration motions. Furthermore, magnetometer measurements can become unreliable indoors. Without magnetometers, however, IMUs lack a heading reference, which leads to unobservability issues. This paper proposes a magnetometer-free estimation method, which provides desirable observability qualities under joint kinematics that sufficiently excite the lower body degrees of freedom. The proposed lower body model expands on the current self-calibrating human-IMU estimation literature and demonstrates a novel knee hinge model, the inclusion of segment length anthropometry, segment cross-leg length discrepancy, and the relationship between the knee axis and femur/tibia segment. The maximum a posteriori problem is formulated as a factor graph and inference is performed via post-hoc, on-manifold global optimization. The method is evaluated (*N* = 12) for a prescribed human motion profile task. Accuracy of derived knee flexion/extension angle (4.34${}^{\circ }$ root mean square error (RMSE)) without magnetometers is similar to current state-of-the-art with magnetometer use. The developed framework can be expanded for modeling additional joints and constraints.

## 1. Introduction

The estimation of the human skeletal pose is a well-studied problem [1,2,3]. The class of techniques referred to as human motion capture, or mocap for short, has been approached through different measurement technologies. While computer vision systems exist [4], the most common approach today is marker-based optical motion capture (OMC) [5]. These techniques generally involve the mounting of reflective markers on the human body which are triangulated in space using a set of specially-equipped cameras. While this approach is very accurate at localizing the position of each marker in the capture volume, additional modeling approaches (for example, OpenSim [6] or the Vicon plugin gait model [7,8]) are required to infer the biomechanical quantities of interest—joint angles, skeletal position, etc. Furthermore, marker-based mocap has measurement limitations. Markers may be occluded or fall off the subject, and the measurement typically must take place in a controlled laboratory environment [9].

Small, wearable inertial measurement units (IMUs) have garnered interest as a low-cost, in-the-field alternative to OMC. These sensor packages typically measure acceleration and angular velocity, and are often augmented with local magnetic field measurements to aid in heading estimation. IMUs have classically been used for inertial navigation on aerospace and nautical systems [10,11,12]. Modern IMUs mounted on the human body are compact and offer motion estimation without external equipment and a line-of-sight requirement. Approaches are developed to overcome the major disadvantages of IMUs, namely system noise and drift of estimated quantities (e.g., [13,14,15,16,17,18]). Along with developed human-body modeling approaches, IMUs have found multiple uses within human studies, including clinical sciences [19,20,21,22,23], sports performance [24,25,26,27,28], activity recognition [29,30,31,32,33], occupational ergonomics [34], and even spacesuit fit evaluation [35].

One major consideration to be made when using IMU-based human modeling approaches is prescribing or estimating the relationship between the IMU coordinate frame on the surface of the body segment and the underlying skeletal system in which many biomechanical quantities of interest are defined, sometimes referred to as the IMU-to-segment (I2S) pose relationship. A recent review by Vitali and Perkins [36] places these methods into four distinct categories—(1) assumed alignment methods, (2) functional alignment methods, (3) model-based methods, and (4) augmented data methods. Assumed alignment approaches have considered the a priori alignment of the IMU to the human skeletal system [37]. Mayagoitia et al. [38] used aligned gyroscope and accelerometer packages to track the pitch angle of the shank to an error of 2.7${}^{\circ }$± 2.8${}^{\circ }$. Favre et al. [39,40] precisely aligns one of the axes of the IMU with the knee’s major rotation axis. Kok et al. [41] formulates the problem as a maximum a posteriori (MAP) estimation problem, solved through weighted least squares optimization, including the estimation of offset vectors from neighboring segment coordinate systems to their common point-revolute joint, after determining the I2S relationship from a priori measurement and a static calibration routine [42]. These methods are limited by the operator’s ability to precisely align the IMU, or their ability to measure the alignment offset. Functional alignment methods have considered using static or dynamic calibrations (referred to as functional calibrations) which the human subject must perform in order to determine the alignment between the IMU and skeletal system. Favre et al. [43,44] prescribes a functional calibration set with a subsequent root mean square error (RMSE) of the knee’s three major rotation angles between 4.0${}^{\circ }$ and 8.1${}^{\circ }$. Many other works have also used functional calibration to aid in the estimation of segment kinematics [45,46,47,48,49,50,51,52]. These methods may be confounded by the subject’s ability to perform the prescribed calibrations.

More recently, efforts have shifted towards overcoming these limitations of functional or static calibrations. In this work, the phrase self-calibrating is used to describe model-based approaches that either (a) estimate the pose relationship between the IMU and underlying bone segment coordinate system (i.e., the I2S relationship), or (b) can derive relevant biomechanical parameters of the human skeleton (such as joint angles) without directly estimating the I2S relationship. These approaches generally exploit kinematic relationships that exist between IMUs mounted on a semi-rigid-body about a fixed-point rotating joint. Müller et al. [53] models the elbow as a 2 degree of freedom (DOF) system, and argues that the magnitude of the relative angular velocity about the joint may be decomposed into axis components through a formulated optimization problem. McGrath et al. [54] extends this argument to the 1DOF knee hinge joint, offering a computationally-efficient axis estimator based on principal component analysis (PCA). Seel et al. [55,56] presents an optimization-based approach to estimating joint axis and position in the IMU frames from measured angular velocities and accelerations for the knee and ankle joints. This method is expanded on by Salehi et al. [57] with a modified measurement model of the IMU positions relative to their neighboring joints to aid in accuracy and observability. Taetz et al. [58] presents a similar problem solved via a sliding-window weighted least squares algorithm, but with the novel addition of assuming a topology on which the IMU is mounted. In that work, the authors assume a capsule-type shape, but note that other shapes can be included. Often, these works estimate or otherwise kinematically include (i.e., through a priori measurement) an I2S calibration parameter for each IMU [41,58,59], typically modeled as a static SE(3) transformation between the underlying limb coordinate system and the coordinate system of the IMU on the limb surface. Other works [53,54,55,56,60,61] seek to derive relevant biomechanical states without directly estimating the underlying skeletal coordinate systems, a potentially minimal parameterization of the problem. The current work falls in the latter category. There are two major motivations for this approach: First, as noted in Taetz et al. [58], to estimate both the IMU coordinate system trajectory and the skeletal coordinate system trajectory (when the I2S relationship is assumed static) is a redundant variable set. Secondly, when mathematical relationships are cast as a function of skeletal coordinate system (rather than the IMU coordinate system), the resultant noise distributions are often immeasurable. For example, as of this publication, the authors are not aware of attempts to measure the noise distribution of (a) bone connection "error" at a single rotation center (i.e., the constraint first proposed by Kok et al. [41] and used by subsequent works [58]) when the constraint is cast in the skeletal coordinate system or (b) the distribution of the aforementioned static I2S parameter in SE(3), although efforts have been made [62] to investigate the sensitivity of the optimized solution to the accuracy of this parameter. This modeling dichotomy deserves continued investigation.

These works also vary in their solution methodology. Many consider general batch optimization [41,53,55,56], while others have considered sliding window approaches [58], filtering approaches [52], or even PCA-based approaches [54]. Recently, Kok et al. [63] reconsiders the optimization problem [41] but with a more efficient approach to the selection of search direction, using a message passing algorithm [64].

Performing batch optimization for this class of problems is expensive due to high-frequency data measured from multiple IMUs. It has been proposed to use keyframes [65] to reduce the size of the optimization problem, i.e., to select only a relevant subset of the full state trajectory for MAP inference. Depending on the application, this keyframe approach can also yield faster solutions without loss in accuracy or operational decision making ability. For example, human walking can be captured with a slower keyframe rate than sprinting, and likely neither require estimated states at the same rate as high frequency IMU samples. Lupton and Sukkarieh [66] first proposed an implementation for inertial sensors as preintegrated IMU measurements. The current work relies on the implementation by Carlone et al. [67] and Forster et al. [68]. Their IMU preintegration work develops a relative motion constraint between keyframes, which robustly handles IMU bias propagation. Moreover, the IMU model variables, as with all relevant variables in this work, are implemented on-manifold for efficient computation (for a background on differential manifolds, Lie groups, and applications to visual/inertial/human navigation problems, the reader is referred to [69,70,71]). The GTSAM 4.0 library [72], which uses factor graphs [73] as the underlying computational framework, is a commonly-used library for on-manifold optimization and includes the IMU preintegration work.

Another consideration is that magnetometers are notoriously unreliable indoors [74,75] due to the presence of electromagnetic disturbances. While some works have considered compensation or recalibration for these disturbances [76,77,78,79], many works, including this one, avoid the use of magnetometers altogether [41,80]. Without the use of magnetometers, IMUs typically do not have a heading reference, leaving the yaw degree of freedom generally unobservable in the case of traditional dead-reckoning for IMU pose trajectory. Therefore, exploiting kinematic constraints inherent to the human skeletal system (cast in local frames, i.e., between body segments) may improve observability [57], however, may not provide absolute observability of the IMU heading angle, i.e., all solutions rotated about the global vertical axis are equivalent. Discerning the heading relationship between two IMUs flanking a joint may be possible using an approach that leverages a model of a static vector pair from the joint to the neighboring IMUs, e.g., [41], however, when these vectors lie normal to the horizontal plane they may not provide sufficient information. Tasks such as upright gait are likely to have static vectors from the IMU to joint centers that are directed proximal or distal on the limb, which would be approximately parallel to the global upward vector. In these situations, more information is necessary to discern the heading relationship between two IMUs. The current work presents a novel knee kinematic model for least-squares optimization which leverages the kinematic relationship between the pair of IMUs’ orientations and angular velocities in order to estimate a static rotation axis in each IMUs’ local frame. In the case of upright gait and an appropriately-behaved kinematic hinge (such as the human knee), the proposed model would provide enough information to discern the heading relationship between the two IMUs flanking the joint center. The direct estimation of the hinge axis, which points medial/lateral in the skeletal frames, permits the definition of segment coordinate systems consistent with the International Society of Biomechanics’ (ISB) recommendations [81,82,83]. Deriving joint angles according to ISB convention has the explicit benefit of both allowing comparison across different methods and presenting angles in a common convention that is already accepted.

In this work, we consider the problem of estimating lower body skeletal kinematics from a requisite set of seven IMUs mounted on the subject (on the lower back, thighs, shanks, and feet) under human motion without a heading reference. The proposed optimization-based approach uses accelerometer and gyroscope measurements to estimate IMU pose trajectories, knee hinge axes statically represented in the thigh and shank IMU local frames, and the assumed-static relationship between the IMU frame and its neighboring joint center(s) subject to a number of kinematic constraints. These estimated parameters allow for post-hoc derivation of the underlying femur, tibial, and pelvic coordinate systems, and as a result, the skeletal joint angles for the knee. A performance of the proposed approach is evaluated for 12 subjects, each performing a prescribed motion profile designed to excite all human lower body degrees of freedom. Additionally, the subject is outfitted with optical motion capture tracking as a truth datum. When appropriate, the proposed method is compared against a control IMU algorithm, which considers each of the seven IMUs individually as a dead-reckoning problem in an optimization-based smoothing framework.

We hypothesize that (1) the proposed model will not significantly affect error of estimated IMU orientations for the traditionally-observable orientation degrees of freedom (pitch and roll) and (2) that the derived knee flexion/extension angle will fall within 5 degrees RMSE when compared to optical motion capture estimates. Additionally, this work will characterize IMU position estimation accuracy.

This work extends the literature for estimation of human limb pose estimation from body-worn IMUs in three key ways. (1) This work presents a novel knee kinematic model similar to McGrath et al. [54] but reformulated for least-squares optimization, additionally exploiting kinematic information about the relationship between the knee’s rotation axis and the femur and tibia proximal direction [84]. (2) This work demonstrates the inclusion of population anthropometric information as a probabilistic constraint, namely length of the femur, tibia, and femoral head separation as well as femur and tibia length discrepancy. (3) This work leverages IMU preintegration theory [67,68] to reduce problem size and complexity without loss in accuracy or relevant estimated information. In addition to these contributions, the MAP estimation problem is presented in factor graph form, an expressive modeling framework which allows for a flexible inclusion of priors and advanced inference techniques. While in the presented problem the kinematic constraints are included at each keyframe, one could easily only include the kinematic constraints at a subset of keyframes or constrain across nonconsecutive keyframes if such information existed. This work adopts recommended conventions by the ISB for reporting knee angles [82] to increase operational interpretation for end users, which is made possible through the estimation of the knee’s rotation axis. The method is tested in a multi-subject study with differences between subjects considered.

## 2. Problem Formulation

**Figure 1:**
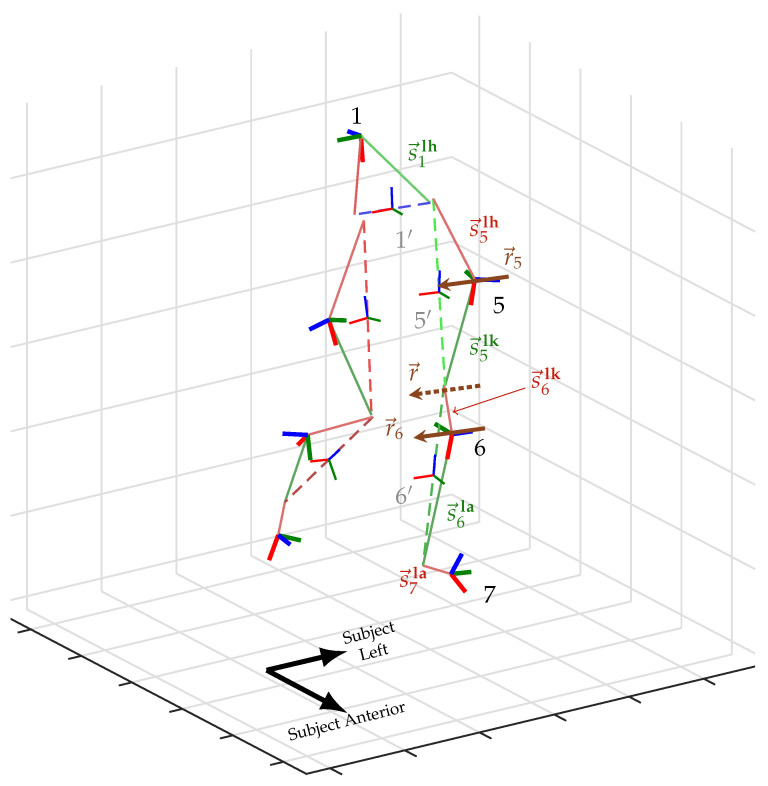
The human-IMU kinematic system, with the subject mid-stride. Image is an excerpt frame from a 3D animation using the proposed method. The subject’s left leg is labeled with coordinate systems of the IMUs (bold RGB triplets with black text label) and anatomical segments (thin RGB triplets with gray text label), the static vectors from the IMUs to neighboring joint centers (red and green), and the knee’s hinge axis (dotted brown) with their static representations in the thigh and shank IMU frames (solid brown). Notation of variables is detailed in Section 2.1.

The method requires a human subject to be outfitted with IMUs on the lower back, thighs, shanks, and feet. The assignment of IMU to limb segment is known however, the placement (i.e., orientation and position) of the IMU on the segment is unknown. It is desired to estimate the time-series pose trajectories of each IMU along with a static biomechanical map which relates these IMUs to the underlying skeletal system to which they are jointly attached. No reference of position or heading angle is assumed to be known—only measurements of the accelerometer and gyroscope of each IMU are taken. A visual representation of this system is shown in Figure 1.

### 2.1. Estimated Variables, Derived Quantities, and Notation

The local coordinate frames of the IMUs mounted on the lumbar, right thigh, right shank, right foot, left thigh, left shank, and left foot are denoted as 1, 2, 3, 4, 5, 6, and 7 respectively. The coordinate frames of the underlying pelvis, right femur, right tibia, right calcaneus, left femur, left tibia, and left calcaneus anatomical segments are denoted with a prime symbol (${}^{\prime }$) above their associated IMU frame: $1^{\prime }$, $2^{\prime }$, $3^{\prime }$, $4^{\prime }$, $5^{\prime }$, $6^{\prime }$, and $7^{\prime }$, respectively. The shared navigation frame is denoted as N. Denote a numbered *keyframe* k=1…M, within the set of all keyframes K. Let i,j∈K denote any two consecutive keyframes. The set of all IMU measurements (gyroscope and accelerometer) between keyframes *i* and *j* is denoted $I_{\left(i,j\right)}$. As explained later (Section 2.2.1), the IMU states are not estimated at each measurement, but rather at a lower rate for computational efficiency.

The following variables are estimated in the model:

- Discrete time-series pose trajectory of each IMU: $X_{1,k}^{N}$, $X_{2,k}^{N}$, $X_{3,k}^{N}$, $X_{4,k}^{N}$, $X_{5,k}^{N}$, $X_{6,k}^{N}$, $X_{7,k}^{N}$∈SE(3) for k=1…M. It should be noted that a pose from frame *A* to the navigation frame, $X_{A}^{N}\in SE\left(3\right)$ may equivalently be expressed in terms of its orientation $R_{A}^{N}\in SO\left(3\right)$ and position $p^{A}\in R^{3}$ components;
- Discrete time-series velocities of each IMU: ${\vec{v}}_{1,k}$, ${\vec{v}}_{2,k}$, ${\vec{v}}_{3,k}$, ${\vec{v}}_{4,k}$, ${\vec{v}}_{5,k}$, ${\vec{v}}_{6,k}$, ${\vec{v}}_{7,k}$∈$R^{3}$ for k=1…M;
- Discrete time-series angular velocities of each IMU: ${\hat{\omega }}_{k}^{1}$, ${\hat{\omega }}_{k}^{2}$, ${\hat{\omega }}_{k}^{3}$, ${\hat{\omega }}_{k}^{4}$, ${\hat{\omega }}_{k}^{5}$, ${\hat{\omega }}_{k}^{6}$, ${\hat{\omega }}_{k}^{7}$$\in R^{3}$ for k=1…M;
- Discrete time-series accelerometer and gyroscope biases for each IMU: ${\vec{b}}_{1,k}$, ${\vec{b}}_{2,k}$, ${\vec{b}}_{3,k}$, ${\vec{b}}_{4,k}$, ${\vec{b}}_{5,k}$, ${\vec{b}}_{6,k}$, ${\vec{b}}_{7,k}$∈$R^{6}$ for k=1…M;
- The (static) hinge axis of the right knee, expressed in the right thigh and right shank IMU frame, respectively: ${\vec{r}}_{2},{\vec{r}}_{3}$∈$S^{2}$, and similar for the (static) axis of the left knee expressed in its respective thigh and shank frame: ${\vec{r}}_{5},{\vec{r}}_{6}$∈$S^{2}$;
- The (static) vector from the IMU frame to each adjacent joint center, i.e., the vector from the
  – lumbar IMU frame to the right hip rotation center: ${\vec{s}}_{1}^{\;\mathrm{rh}}\in R^{3}$,
  – right thigh IMU to the right hip center: ${\vec{s}}_{2}^{\;\mathrm{rh}}\in R^{3}$,
  – right thigh IMU to the right knee center: ${\vec{s}}_{2}^{\;\mathrm{rk}}\in R^{3}$,
  – right shank IMU to the right knee center: ${\vec{s}}_{3}^{\;\mathrm{rk}}\in R^{3}$,
  – right shank IMU to the right ankle center: ${\vec{s}}_{3}^{\;\mathrm{ra}}\in R^{3}$,
  – right foot IMU to the right ankle center: ${\vec{s}}_{4}^{\;\mathrm{ra}}\in R^{3}$
  – lumbar IMU frame to the left hip rotation center: ${\vec{s}}_{1}^{\;\mathrm{lh}}\in R^{3}$,
  – left thigh IMU to the left hip center: ${\vec{s}}_{5}^{\;\mathrm{lh}}\in R^{3}$,
  – left thigh IMU to the left knee center: ${\vec{s}}_{5}^{\;\mathrm{lk}}\in R^{3}$,
  – left shank IMU to the left knee center: ${\vec{s}}_{6}^{\;\mathrm{lk}}\in R^{3}$,
  – left shank IMU to the left ankle center: ${\vec{s}}_{6}^{\;\mathrm{la}}\in R^{3}$,
  – left foot IMU to the left ankle center: ${\vec{s}}_{7}^{\;\mathrm{la}}\in R^{3}$.

The following quantities are not estimated, but rather derived from estimated variables in the model:

- Discrete time-series orientations of the anatomical pelvic, right femur, right tibial, left femur, and left tibial segments: $R_{1^{\prime },k}^{N}$, $R_{2^{\prime },k}^{N}$, $R_{3^{\prime },k}^{N}$, $R_{5^{\prime },k}^{N}$, $R_{6^{\prime },k}^{N}\in SO\left(3\right)$ for k=1…M;
- The time-series flexion/extension, internal/external rotation, and abduction/adduction joint angles of the knee.

The coordinate systems of the pelvis, femur, and tibia segments are assumed to be statically related to the coordinate systems of the IMU mounted on the surface of the associated limb segment. These anatomical coordinate systems are defined according to the recommendations of the International Society of Biomechanics ([82]):

*z*: positive in the proximal direction;
*y*: positive in the anterior direction;
*x*: positive to the subject’s right.

This choice of the anatomical coordinate system is motivated by the desire to derive joint angle definitions that are meaningful to end users.

### 2.2. Model

The following sections detail the proposed model of this work. Section 2.2.1 describes the time-series IMU dynamics, Section 2.2.2, Section 2.2.3, Section 2.2.4, Section 2.2.5, Section 2.2.6, and Section 2.2.7 describe the biomechanical and anthropometric constraints: Knee pseudo-hinge kinematics (Section 2.2.2), the relationship between the knee’s hinge axis and the femur/tibia proximal direction (Section 2.2.4), constrained joint center of rotation between adjacent IMUs (Section 2.2.3), anthropometry of femur length, tibia length, and pelvic width (Section 2.2.5), maximum/minimum allowable anthropometric lengths (Section 2.2.6), and femur and tibia length discrepancy (Section 2.2.7). Section 2.2.8 presents this model within a MAP estimation framework. Finally, Section 2.2.9 presents noteworthy model unobservability conditions.

#### 2.2.1. IMU Dynamics Model

The following IMU dynamics model is due to Forster et al. [68] and summarized here. An IMU takes measurements in the local body frame *B*, specifically the rotation rate about the local body axes, $\tilde{\omega }$, and body acceleration in the local frame $\tilde{a}$. These measurements are imperfect, corrupted by slowly-varying biases ${\vec{b}}^{\;\omega }$ and ${\vec{b}}^{a}$, and additive zero-mean white noise $v_{\omega }∼N\left(0,\sigma_{\omega }^{2}\right)$ and $v_{a}∼N\left(0,\sigma_{a}^{2}\right)$.

(1a) $$\tilde{\omega }\left(t\right)=\omega \left(t\right)+{\vec{b}}^{\;\omega }\left(t\right)+v_{\omega }\left(t\right)$$

(1b) $$\tilde{a}\left(t\right)={\left(R_{B}^{N}\right)}^{-1}\left(a_{N}\left(t\right)-g\right)+{\vec{b}}^{a}\left(t\right)+v_{a}\left(t\right)$$

where g is the acceleration due to gravity constant, expressed in the navigation frame. Using a kinematic model:

(2) $$\frac{dR_{B}^{N}\left(t\right)}{dt}=R_{B}^{N}\left(t\right){\left[\omega (t)\right]}_{\times },\frac{d\vec{v}\left(t\right)}{dt}=a_{N}\left(t\right),\frac{dp^{}\left(t\right)}{dt}=\vec{v}\left(t\right)$$

and integrating over the interval [t,t+Δt], the kinematic model as a function of the IMU measurements $\tilde{\omega }$ and $\tilde{a}$ is written:

(3a) $$R_{B}^{N}\left(t+\Delta t\right)=R_{B}^{N}\left(t\right)\exp\left(\left(\tilde{\omega }\left(t\right)-{\vec{b}}^{\;\omega }\left(t\right)-v_{\omega }^{d}\left(t\right)\right)\Delta t\right)$$

(3b) $$\vec{v}\left(t+\Delta t\right)=\vec{v}\left(t\right)+g\Delta t+R_{B}^{N}\left(t\right)\left(\tilde{a}\left(t\right)-{\vec{b}}^{a}\left(t\right)-v_{a}^{d}\left(t\right)\right)\Delta t$$

(3c) $$p^{}\left(t+\Delta t\right)=p^{}\left(t\right)+\vec{v}\left(t\right)\Delta t+\frac{1}{2}g\Delta t^{2}+\frac{1}{2}R_{B}^{N}\left(t\right)\left(\tilde{a}\left(t\right)-{\vec{b}}^{a}\left(t\right)-v_{a}^{d}\left(t\right)\right)\Delta t^{2}$$

where $v_{a}^{d}\left(t\right)$ is the discrete-time noise, related to the continuous-time spectral noise through $Cov\left(v_{a}^{d}\left(t\right)\right)=\frac{1}{\Delta t}Cov\left(v_{a}\left(t\right)\right)$, and similarly for $v_{\omega }^{d}\left(t\right)$.

Equation (3) represent a valid set of constraints that could be incorporated in an inference problem to model IMU dynamics and estimate the state trajectory of an IMU $\left\{R\left(t\right),p^{}\left(t\right),\vec{v}\left(t\right),{\vec{b}}^{a}\left(t\right),{\vec{b}}^{\;\omega }\left(t\right)\right\}$. However, typically these equations are discretized at the the same rate as the IMU measurements (i.e., $\frac{1}{\Delta t}$) which is often more frequent than necessary for most biomechanics applications. Forster et al. [68] develops a suitable model for IMU preintegration on-manifold, which enables the summary of all IMU accelerometer and gyroscope measurements between two keyframes k=i and k=j, denoted by $I_{\left(i,j\right)}$, into a single compound probabilistic constraint. Given $I_{\left(i,j\right)}$, the residual error model $r_{I_{i,j}}\in R^{9}$ and the error model of slowly-time-varying measurement bias $r_{b_{i,j}}\in R^{6}$ are given in Equations (37) and (40) in Forster et al. [68]. This model of IMU dynamics evolution is used for each IMU in the current work.

Additionally, the instantaneous angular velocity of the IMU at keyframe *k* is also estimated. The estimated angular velocity $\hat{\omega }$ is simply related to the gyroscope measurement $\tilde{\omega }$ which coincides with keyframe *k* as:

(4) $$e_{k}=f_{\omega }\left({\hat{\omega }}_{k},{\vec{b}}_{k}^{\;\omega },{\tilde{\omega }}_{k}\right)={\hat{\omega }}_{k}+{\vec{b}}_{k}^{\;\omega }-{\tilde{\omega }}_{k},\;e_{k}∼N\left(0,\Sigma_{g}\right)\;\forall \;k.$$

#### 2.2.2. Knee Pseudo-Hinge Kinematics

**Figure 2:**
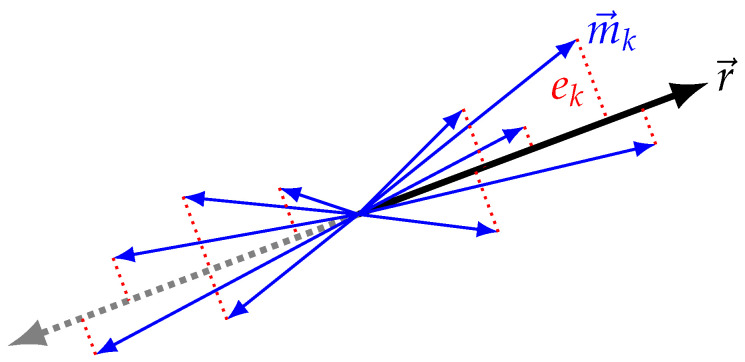
The set of relative angular velocity vectors ${\vec{m}}_{k}$ projected onto knee axis $\vec{r}$ with resulting residuals $e_{k}$. Note that for a perfect hinge $m_{k}\|\vec{r}\;\forall \;k$ however, due to imperfect hinge kinematics of the human knee and soft tissue perturbation of the gyroscopes mounted to the skin of the leg, the set of vectors ${\vec{m}}_{k}$ takes this characteristic double cone shape.

The human knee is assumed to act as a pseudo-hinge with a single point of rotation. Moreover, the major hinge axis $\vec{r}$ is assumed to be statically representable in both the thigh IMU and shank IMU coordinate frames. For a perfect point-rotation hinge joint, the relative angular velocity across the joint will point normal to the plane of rotation (see McGrath et al. [54]). At every estimated IMU state keyframe, this relative angular velocity ${\vec{m}}_{k}$ (Figure 2) may be computed. Due to soft tissue artifacts and the imperfect hinge kinematics of the joint, these vectors will not all point exactly in the same direction.

A point estimate of the hinge axis $\vec{r}$ may be found by minimizing the projection of ${\vec{m}}_{k}$ onto $\vec{r}$, as given by:

(5) $$\frac{\left({\vec{m}}_{k}\;\cdot \;\vec{r}\right)\vec{r}}{{∥\vec{r}∥}_{2}^{2}}\;\forall \;k.$$

Since $\vec{r}$ is always a unit axis, ${∥\vec{r}∥}_{2}^{2}=1$. Hence, the line segment between ${\vec{m}}_{k}$ and the projection of ${\vec{m}}_{k}$ onto $\vec{r}$ is simplified to ${\vec{m}}_{k}-\left({\vec{m}}_{k}\;\cdot \;\vec{r}\right)\vec{r}$. The error model is then given by:

(6) $$e_{k}={\vec{m}}_{k}-\left({\vec{m}}_{k}\;\cdot \;\vec{r}\right)\vec{r},\;e_{k}∼N\left(0,\Sigma_{k}\right)\;\forall \;k.$$

Consider a hinge joint flanked by an IMU proximal and distal to the joint, *A* and *B* respectively. Then for each keyframe *k*, the relative angular velocity across the joint may be computed in frame *A* as:

(7) $$m_{k}={\vec{\omega }}_{B-A}^{A}={\left(R_{A,k}^{N}\right)}^{-1}R_{B,k}^{N}\;{\hat{\omega }}_{B,k}-{\hat{\omega }}_{A,k}\;\forall \;k.$$

Combining Equation (6) and (7), the full error model for the hinge axis expressed statically in frame *A* may be rewritten as:

(8) $$\begin{array}{cc} \; & \;e_{k}=f_{K}\left({\vec{r}}_{A},R_{A,k}^{N},R_{B,k}^{N},{\hat{\omega }}_{A,k},{\hat{\omega }}_{B,k}\right)=\\ \; & {R_{A,k}^{N}}^{-1}R_{B,k}^{N}{\hat{\omega }}_{B,k}-{\hat{\omega }}_{A,k}-\left({R_{A,k}^{N}}^{-1}R_{B,k}^{N}{\hat{\omega }}_{B,k}-{\hat{\omega }}_{A,k}\;\cdot \;{\vec{r}}_{A}\right){\vec{r}}_{A}\;e_{k}∼N\left(0,\Sigma_{K}\right)\;\forall \;k. \end{array}$$

This kinematic relationship can also be exploited for the hinge axis in the distal IMU frame by simply rearranging the arguments of Equation (8),

(9) $$e_{k}=f_{K}\left({\vec{r}}_{B},R_{B,k}^{N},R_{A,k}^{N},{\hat{\omega }}_{B,k},{\hat{\omega }}_{A,k}\right)\;\forall \;k.$$

#### 2.2.3. Constrained Joint Centers of Rotation

Originally proposed by Kok et al. [41] and commonly adapted across other implementations, the pose relationship between IMUs can be exploited. For any two IMUs mounted on either side of a 3DOF point-rotation joint, it is assumed that there is a vector which is static in each IMU frame which points to the center of rotation of the common joint. In the navigation frame, these vectors, composed with their associated IMU poses, should represent the same point—the joint’s center.

For any IMUs *A* and *B* flanking joint rotation center j, there exist static vectors in each IMU frame (${\vec{s}}_{A}^{\;j}$ and ${\vec{s}}_{B}^{\;j}$ respectively), such that the following relationship holds:

(10) $$e_{k}=f_{J}\left(X_{A,k}^{N},{\vec{s}}_{A}^{\;j},X_{B,k}^{N},{\vec{s}}_{B}^{\;j}\right)=X_{A,k}^{N}{\vec{s}}_{A}^{\;j}-X_{B,k}^{N}{\vec{s}}_{B}^{\;j},\;e_{k}∼N\left(0,\Sigma_{j}\right)\;\forall \;k.$$

#### 2.2.4. Angle Between the Knee Rotation Axis and Femur/Tibia Proximal

There exists an anthropometric relationship between the knee’s primary axis of rotation and the femur/tibia segment length. Hollister et al. [84] measured this angular distribution between the knee’s (lateral-pointing) rotation axis and the femur and tibia segments through the range of knee flexion/extension, and this relationship is not necessarily orthogonal. Such a model is useful in aiding model identifiability, discussed in detail in Section 2.2.9. A probabilistic constraint can be formulated to model this relationship. The angle between an axis $a\in R^{3}$ and a segment *s*, both expressed in a common frame, can be written as:

(11) $$\theta =\arccos\left(\frac{a\;\cdot \;s}{\left({∥a∥}_{2}{∥s∥}_{2}\right)}\right)$$

where it is noted that for a unit axis, ${∥a∥}_{2}=1$. This motivates the following error model for the right thigh:

(12) $$e=f_{o}\left({\vec{r}}_{2},{\vec{s}}_{2}^{\;\mathrm{rh}},{\vec{s}}_{2}^{\;\mathrm{rk}}\right)=\mu -\arccos\left(\frac{{\vec{r}}_{2}\;\cdot \;\left({\vec{s}}_{2}^{\;\mathrm{rh}}-{\vec{s}}_{2}^{\;\mathrm{rk}}\right)}{{∥{\vec{s}}_{2}^{\;\mathrm{rh}}-{\vec{s}}_{2}^{\;\mathrm{rk}}∥}_{2}}\right),\;e∼N\left(0,\sigma_{o}^{2}\right)$$

and similarly for other segments, where μ and $\sigma_{o}$ are the expected mean and standard deviation of the angle between the knee’s rotation axis and the segment proximal vector. In Hollister et al. [84], the values for μ are given for the exterior angle between the medial-pointing knee rotation axis and femur and tibia. However, in the proposed model, the knee’s rotation axis is always assumed to point to the subject’s right per ISB conventions [82]. These angular relationships can be adjusted through simple geometric identities. The adjusted values for μ and $\sigma_{o}$ can be found in Table 1.

#### 2.2.5. Femur Length, Tibia Length, and Pelvic Width from Anthropometry

For any IMU that is between two neighboring joint centers, i.e., the thigh and shank IMUs, there exists an anthropometric relationship between these two joint centers, which has likely been tabulated in an anthropometric database. This relationship more precisely specifies the kinematic model of the human, especially under insufficient kinematics to identify model parameters or significant measurement noise. For an IMU *A* between two joints centers $j_{1}$ and $j_{2}$, the static vectors from the IMU frame to the neighboring joint frame would be denoted ${\vec{s}}_{A}^{\;j_{1}}$ and ${\vec{s}}_{A}^{\;j_{2}}$. Let the distance between these joint centers be denoted *L*, where an approximately-normal distribution from anthropometric measurements exists as $L∼N(\mu_{L},\sigma_{L}^{2})$. Then the distance between joint centers $j_{1}$ and $j_{2}$ may be constrained using this prior information:

(13) $$\begin{array}{c} e=f_{L}\left({\vec{s}}_{A}^{\;j_{1}},{\vec{s}}_{A}^{\;j_{2}};\mu_{L}\right)={∥{\vec{s}}_{A}^{\;j_{1}}-{\vec{s}}_{A}^{\;j_{2}}∥}_{2}-\mu_{L},\;e∼N\left(0,\sigma_{L}^{2}\right). \end{array}$$

#### 2.2.6. Maximum/Minimum Anthropometric Lengths

In addition to the probabilistic encoding of subject anthropometry in Equation (13), it is sometimes also useful to encode a hard maximum and/or minimum anthropometric length, which practically limits the solution space to reasonable values of segment lengths. This constraint is specifically relevant when using generic population measures for anthropometry (as is done in this work) rather than specifically calibrated anthropometric lengths. For a given state variable *x* for which minimum $x_{min}$ and maximum $x_{max}$ are desired, a continuously differentiable function may be defined which models zero error on the interval $\left[x_{min},x_{max}\right]$, with increasing error otherwise.

(14) $$f_{M}\left(x;x_{min},x_{max},a\right)=\left\{\begin{array}{cc} \left(x-x_{max}\right){\left(\tanh\left(a\left(x-x_{max}\right)\right)\right)}^{2} & x>x_{max}\\ \left(x_{min}-x\right){\left(\tanh\left(a\left(x_{min}-x\right)\right)\right)}^{2} & x<x_{min}\\ 0 & \mathrm{otherwise} \end{array}\right.$$

This may be encoded as a noise model in the system. For any vectors in frame *A* which span a common limb segment and anthropometric limits $x_{min}$ and $x_{max}$,

(15) $$e=f_{M}\left({∥{\vec{s}}_{A}^{\;j_{1}}-{\vec{s}}_{A}^{\;j_{2}}∥}_{2},x_{min},x_{max};a\right).$$

As this error model is introduced to the system to model a hard limit constraint, the distribution of *e* is not motivated from the dynamics of the system. Rather, the “strength” of the constraint may be tuned through the adjustment of error model scalar parameter *a*.

#### 2.2.7. Femur and Tibia Length Discrepancy

In addition to aforementioned anthropometric information about the distribution of femur and tibia lengths, there also exists anthropometric information about the discrepancy, or difference, between the right and left femur and tibia [85,86,87]. Similarly, such a constraint is useful to precisely specify the human kinematic model, especially under insufficient kinematics or high measurement noise. Assuming that this distribution is on average zero for the greater population, a probabilistic model of this error distribution for the femur is written:

(16) $$e=f_{D}\left({\vec{s}}_{2}^{\;\mathrm{rh}},{\vec{s}}_{2}^{\;\mathrm{rk}},{\vec{s}}_{5}^{\;\mathrm{lh}},{\vec{s}}_{5}^{\;\mathrm{lk}}\right)={∥{\vec{s}}_{2}^{\;\mathrm{rh}}-{\vec{s}}_{2}^{\;\mathrm{rk}}∥}_{2}-{∥{\vec{s}}_{5}^{\;\mathrm{lh}}-{\vec{s}}_{5}^{\;\mathrm{lk}}∥}_{2},\;e∼N\left(0,\sigma_{D}^{2}\right)$$

and similarly for the tibia, where $\sigma_{D}$ represents the standard deviation of the discrepancy distribution. According to Shultz and Nguyen [85], $\sigma_{D}=0.8$ cm for the femur and $\sigma_{D}=0.6$ cm for the tibia.

#### 2.2.8. Full Problem Representation

Recall the convenient IMU numbering scheme of the IMU on the lower back, right thigh, right shank, right foot, left thigh, left shank, and left foot as S={1,…,7} respectively. Let IMU coordinate frames *A* and *B* and joint center(s) j represent relevant parameters of any of the formulated factor error models above. The posterior probability of all variables Y given all measurements Z and priors $p(Y_{0})$ is represented as:

(17) $$\begin{array}{cc} \; & p\left(Y|Z\right)\propto p\left(Z|Y\right)p\left(Y_{0}\right)=p\left(Y_{0}\right)\underset{\text{IMU dynamics (Section 2.2.1)}}{\underset{︸}{\prod_{(i,j)\in K}\prod_{s\in S}p\left(I_{\left(i,j\right)}^{s}|X_{s,i}^{N},X_{s,j}^{N}\right)}}\;\underset{\text{IMU angular velocities (Section 2.2.1)}}{\underset{︸}{\prod_{k\in K}\prod_{s\in S}p\left({\tilde{\omega }}_{s,k}|{\hat{\omega }}_{s,k},{\vec{b}}_{s,k}^{\;\omega }\right)}}\\ \; & \;\underset{\text{knee hinge dynamics (Section 2.2.2)}}{\underset{︸}{\prod_{k\in K}\prod_{(A,B)\in S}p\left({\vec{r}}_{A},X_{A,k}^{N},X_{B,k}^{N},{\hat{\omega }}_{A,k},{\hat{\omega }}_{B,k}\right)}}\;\underset{\text{joint connection (Section 2.2.3)}}{\underset{︸}{\prod_{k\in K}\prod_{(A,B)\in S}p\left(X_{A,k}^{N},X_{A,k}^{N},{\vec{s}}_{A}^{\;j},{\vec{s}}_{B}^{\;j}\right)}}\;\underset{\text{knee axis vs. segment proximal (Section 2.2.4)}}{\underset{︸}{\prod_{k\in K}\prod_{A\in S}p\left({\vec{r}}_{A},{\vec{s}}_{A}^{\;j_{1}},{\vec{s}}_{A}^{\;j_{2}}\right)}}\\ \; & \;\underset{\text{Anthropometry (Section 2.2.5)}}{\underset{︸}{\prod_{A\in S}p\left({\vec{s}}_{A}^{\;j_{1}},{\vec{s}}_{A}^{\;j_{2}}\right)}}\;\underset{\text{Segment length discrepancy (Section 2.2.7)}}{\underset{︸}{\prod_{(A,B)\in S}p\left({\vec{s}}_{A}^{\;j_{1}},{\vec{s}}_{A}^{\;j_{2}},{\vec{s}}_{B}^{\;j_{3}},{\vec{s}}_{B}^{\;j_{4}}\right)}} \end{array}$$

The setting of all variables which maximize Equation (17) is referred to as the maximum a posteriori estimate of Y, denoted ${\hat{Y}}_{\mathrm{MAP}}$. Equivalently, ${\hat{Y}}_{\mathrm{MAP}}$ minimizes the negative log likelihood of Equation (17), additionally separating the factors into a simple sum of squares of the residual error models presented in Section 2.2. Under the assumption of Gaussian factors,

(18) $$\begin{array}{cc} \; & {\hat{Y}}_{\mathrm{MAP}}=\underset{Y}{arg max}p\left(Y|Z\right)=\underset{Y}{arg min}\;\left[-\log_{e}\;p\left(Y|Z\right)\right]=\sum_{Y_{0}}{∥e_{0}∥}_{\Sigma_{0}}^{2}+\sum_{s\in S}\sum_{(i,j)\in K}{∥e_{I}^{s}∥}_{\Sigma_{I}}^{2}+\\ \; & \;\sum_{k\in K}\sum_{s\in S}{∥f_{\omega }\left({\hat{\omega }}_{s,k},{\vec{b}}_{s,k}^{\;\omega },{\tilde{\omega }}_{s,k}\right)∥}_{\Sigma_{g}}^{2}+\sum_{k\in K}\sum_{(A,B)\in S}{∥f_{K}\left({\vec{r}}_{A},X_{A,k}^{N},X_{B,k}^{N},{\hat{\omega }}_{A,k},{\hat{\omega }}_{B,k}\right)∥}_{\Sigma_{K}}^{2}+\\ \; & \;\sum_{k\in K}\sum_{(A,B)\in S}{∥f_{J}\left(X_{A,k}^{N},X_{B,k}^{N},{\vec{s}}_{A}^{\;j},{\vec{s}}_{B}^{\;j}\right)∥}_{\Sigma_{J}}^{2}+\sum_{k\in K}\sum_{A\in S}f_{o}{\left({\vec{r}}_{A},{\vec{s}}_{A}^{\;j_{1}},{\vec{s}}_{A}^{\;j_{2}}\right)}^{2}/\sigma_{o}^{2}\\ \; & \;\sum_{A\in S}f_{L}{\left({\vec{s}}_{A}^{\;j_{1}},{\vec{s}}_{A}^{\;j_{2}}\right)}^{2}/\sigma_{L}^{2}+\sum_{(A,B)\in S}f_{D}{\left({\vec{s}}_{A}^{\;j_{1}},{\vec{s}}_{A}^{\;j_{2}},{\vec{s}}_{B}^{\;j_{3}},{\vec{s}}_{B}^{\;j_{4}}\right)}^{2}/\sigma_{D}^{2} \end{array}$$

**Figure 3:**
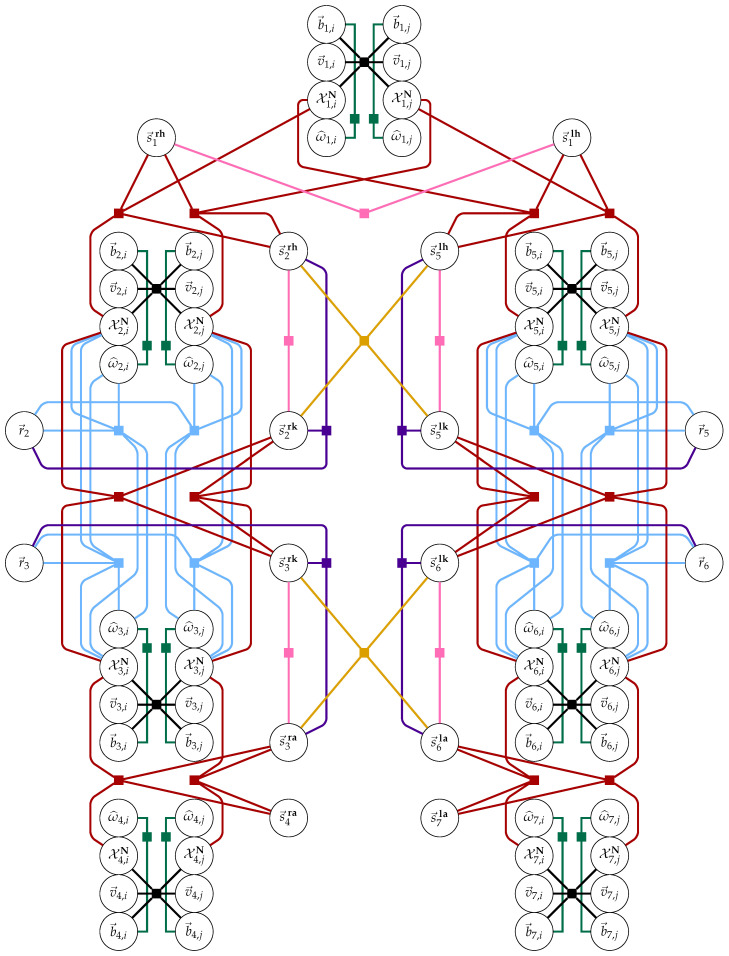
Factor graph representation of the problem for consecutive keyframes *i* and *j*. Variables are represented as circles, whereas the connecting factors are represented as solid squares. For readability, factors and their connecting lines to variables are colored according to factor type: black is the IMU dynamics factor, teal is the angular velocity model, pink is anthropometry, blue is knee hinge kinematics, red is the constrained joint center between IMUs model, violet is the knee axis to segment length quasi-orthogonality factor, and orange is the segment length discrepancy factor. All variable notation is defined in Section 2.1.

The factor graph representation of this problem is given in Figure 3.

#### 2.2.9. Model Identifiability

Following the terminology of Raue et al. [88], the proposed model has a short list of nonidentifiability conditions. There are two structural nonidentifiabilities, i.e., inherent unobservability properties of the model:

(Structural nonidentifiability #1) *Gauge freedom* [89] of the solution in absolute position, velocity, and heading. The proposed model does not have an absolute reference for position, velocity, or heading (i.e., GPS or magnetometers). Therefore, the estimated solution is correct up to a constant offset in these degrees of freedom. This nonidentifiability is addressed through the use of priors to anchor the solution, as detailed in Section 3.7;
(Structural nonidentifiability #2) Knee axis sign ambiguity: Both the positive and negative sign of knee axes ${\vec{r}}_{A}$ and ${\vec{r}}_{B}$ are equivalent nonunique solutions to Equations (8) and (9), respectively. This manifests as $2^{2}=4$ discrete equivalent-error local minima per leg. These equivalent local minima are disambiguated post-hoc, detailed in Section 3.9.

Additionally, the model admits two practical nonidentifiabilities, i.e., nonidentifiabilities which are emergent due to the insufficient quality or amount of sensor observations:

(Practical nonidentifiability #1) (a) A trivial nonidentifiability of static vector and knee axis variables occurs when there is no motion of the subject—the proposed method does require human motion. (b) Similarly, static vectors to the hip and ankle joints must sufficiently explore all DOF of the joints. The solution to the constrained joint center of rotation model (Equation (10)) is only identifiable and unique when both IMUs flanking the joint sufficiently rotate in multiple DOF relative to the joint center;
(Practical nonidentifiability #2) Discerning heading relationship between IMUs flanking the hip and ankle joints. In a magnetometer-free estimation framework, the heading relationship between IMUs must be derived from human kinematics alone. It is possible the constrained joint center of rotation model Equation (10) provides the necessary information. However, in conditions where one or more of the static vectors from the IMU to neighboring joint centers is generally vertical, i.e., orthogonal to the heading plane, then the associated IMU’s orientation trajectory becomes underconstrained and all constant-offset heading solutions are viable. This situation may occur, for example, in upright walking gait with small step length. In the case of the 1DOF knee, this heading relationship between thigh and shank IMUs is well defined by hinge model Equation (8).

The relationship between the knee’s axis and segment direction (Equation (12)) prevents an additional structural nonidentifiability condition from emerging. As previously discussed in practical nonidentifiability #1, both IMUs flanking a joint must sufficiently rotate in multiple DOF relative to the joint center to yield a unique solution to the constrained joint center of the rotation model (Equation (10)). In the case of the 1DOF knee, this is biomechanically impossible. Therefore there would be no unique solution to Equation (10)—the set of solutions would define a knee rotation center anywhere along the medial/lateral line which contains the true knee rotation center. Indeed, for the hip, the medial/lateral location of the rotation center can only be specified through abduction/adduction of the hip. This illustrates the importance of modeling the angular relationship between knee’s axis and the femur/tibia segment length through Equation (12). This model specifies where along the medial/lateral line the knee rotation center must lie to be consistent with human anthropometric data.

## 3. Materials and Methods

### 3.1. Participants

A total of 12 subjects (4 male, 8 female, age = 24.6 ± 3.0 years) participated in this study. The protocol was approved by the Committee on the Use of Humans as Experimental Subjects at MIT (Protocol 1906898310). Exclusion criteria included (1) diagnosis or self-reporting of any lower extremity impairments that limit the subject’s ability to walk, (2) diagnosed or self-reported health conditions (e.g., muscle, heart, etc.) that would prevent the subject for walking for 30 min, (3) inability to walk independently of an assistive device for 30 min on a treadmill, and (4) lack of fluency in the English language.

### 3.2. Study Protocol

Each participant in the study performed a simple motion profile. Subjects additionally walked for 30 min on a treadmill, but those data are not presented in this paper. The motion profile was chosen to excite all degrees of freedom of the lower body, and was performed as follows:

1. *Ankle calibration*: Lift your right foot so that it is hovering a few inches off the ground. Perform three ankle flexion/extension cycles within maximum range of comfort. Then, while foot is lifted a few inches off ground, rotate the front of your foot in a circle three times within maximum range of comfort. Repeat for left ankle;
2. *Knee calibration*: Stand on left foot while keeping both thighs as vertical as possible. Swing right foot behind you (flexing the knee), at least 90 degrees, then return right foot to ground (extending the knee). Do this three times. Repeat for left knee;
3. *Hip calibration*: From standard pose, while keeping knee and ankle neutral, swing your straight right leg up in front of you to maximum range of comfort and return to ground (flexion/extension of hip) three times. Then, perform an adduction/abduction of hip by swinging straight right leg out to lateral side of the body to maximum range of comfort and then returning foot to ground three times. Finally, perform internal/external rotation of hip by keeping foot near to ground and rotating your foot in and out three times to maximum range of comfort while keeping your ankle and knee stiff. Repeat for left hip;
4. *Torso calibration*: From neutral pose, bend down and touch your toes and come back up. Then, twist your torso (forward-left torso twist-forward-right torso twist-forward) to maximum range of comfort. Finally, a side-to-side bend: Up-left-up-right-up.

**Figure 4:**
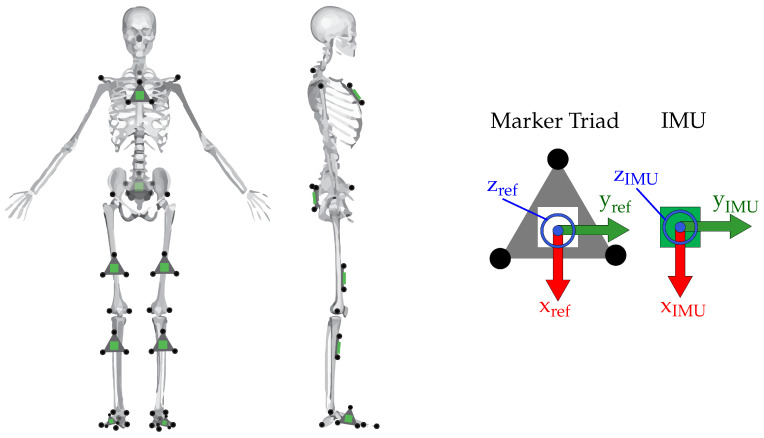
(**Left**) Placement of the reflective markers (black circles) and IMUs (green squares) on the subject. IMUs on the thigh and shank were not placed precisely, and location varied both vertically and in the transverse plane. (**Right**) A blown-up illustration of the marker triads with three markers affixed and IMU. Coordinate system of the IMU was known a priori, and the comparison reference coordinate system of the marker triad was constructed to match.

Each subject was outfitted with a set of reflective motion capture markers (Figure 4) and strap-on IMUs (Opal IMU, APDM, Inc., Portland, OR, USA). The IMUs contained dual 3-axis accelerometers (±16 g, ±200 g), and a 3-axis gyroscope (±2000 deg/s). Markers were placed on anatomical landmarks and on the IMUs. These anatomical landmark markers were placed according to a modified Cleveland Clinic lower body marker set for use in OpenSim [6,90] inverse kinematic modeling. The position of the reflective markers was captured using a 13-camera Vicon motion capture system (Vicon Motion Systems, Inc., Los Angeles, CA, USA) at a sampling rate of 200 Hz. A timing pulse was used to start and stop the recording of the IMU and motion capture data at the same time, so the measurements were aligned in time and of the same length.

### 3.3. Data Processing

**Figure 5:**
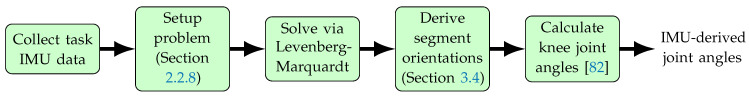
Conceptual process methodology to compute IMU-derived joint angles for the human motion profile task. Levenberg–Marquardt is used as an iterative solver to the proposed optimization problem.

A methodology flowchart illustrating the derivation of IMU-based joint angles for the motion profile task is shown in Figure 5. The optimization of Equation (18) was implemented within the GTSAM 4.0 library [72] in C++ and MATLAB. The measured IMU data were recorded at 200 Hz, and 20 measurements were preintegrated between keyframes, i.e., the time-series IMU poses, velocities, biases—and additionally the derived joint angle calculations—were estimated at 10 Hz. Each of these datasets were processed post-hoc via Levenberg–Marquardt [91]. The size of the system squared Jacobian $J^{⊺}J$ grows quickly: The number of rows or columns is equal to the total size of all variables in the problem (in their manifold representation). For 30 s of data with IMU states estimated at 10 Hz along with static variables for joint hinge axes $\vec{r}$ and static vectors from IMUs to joint centers ${\vec{s}}_{}^{\;}$ this yields a square matrix of size 37,850. The problem was solved on an Intel^®^ Core^™^ i7-4910 MQ CPU (2.90 GHz). Criteria for convergence were the following: An absolute change in error between iterations of 1 × 10^−6^ or less, a relative change in error of 1 × 10^−4^ or less, or 10,000 iterations, whichever was satisfied first. The optical motion capture data, used as a comparison reference in this study, were compared directly to the derived IMU states at 10 Hz. The motion capture data were low-pass filtered with a 30 Hz, 6th order Butterworth filter and then processed in OpenSim 4.0 [6,90], with inverse kinematics computed according to OpenSim’s gait 2392 model [92,93,94,95]. A custom subject model was constructed for each subject prior to processing in the OpenSim solver by scaling the generic model according to anthropometric measures derived from the subject’s marker data while static. Additionally, the 3-marker acrylic reference placed on each IMU (right side of Figure 4) allowed for the simple calculation of a comparison reference for IMU orientation and position in the optical motion capture frame. The IMU data were processed according to the proposed model in Section 2. For each of the 12 motion trials, time-series measurements were compared: Knee angles as derived in Section 3.4 vs. the OpenSim-estimated joint angles and estimated IMU orientation/position vs. marker-based orientation/position comparison reference. It should be noted that optical motion capture is also an imperfect measurement system however, it is still useful to understand the comparison of IMU-derived measures against accepted gold standard technologies.

### 3.4. Derivation and Processing of Knee Angles

From the estimated states, it is desired to derive coordinate systems of the underlying femur, tibia, and pelvic anatomical segments. The knee axis and the proximal direction of the femur and tibia are not necessarily orthogonal [84]. The following function is used to construct an orthonormal leg coordinate system (LCS) where the proximal direction (+z) is fixed and the right direction (+x) is corrected to ensure orthonormality, with the anterior direction (+y) derived from the cross product:

(19) $$R_{A}^{A^{\prime }}=f_{LCS}\left(x,z\right)=\left[\begin{array}{cc} \; & {\left(\left(z\times x\right)\times z\right)}^{⊤}\\ \; & {\left(z\times x\right)}^{⊤}\\ \; & z^{⊤} \end{array}\right].$$

Then, the orientation between the IMU on the leg segment and underlying anatomical segment can be derived for the right femur, right tibia, left femur, and left tibia from the relevant knee axis and normalized proximal vector,

(20a) $$R_{2}^{2^{\prime }}=f_{LCS}\left({\vec{r}}_{2},\frac{\left({\vec{s}}_{2}^{\;\mathrm{rh}}-{\vec{s}}_{2}^{\;\mathrm{rk}}\right)}{{∥{\vec{s}}_{2}^{\;\mathrm{rh}}-{\vec{s}}_{2}^{\;\mathrm{rk}}∥}_{2}}\right)$$

(20b) $$R_{3}^{3^{\prime }}=f_{LCS}\left({\vec{r}}_{3},\frac{\left({\vec{s}}_{3}^{\;\mathrm{rk}}-{\vec{s}}_{3}^{\;\mathrm{ra}}\right)}{{∥{\vec{s}}_{3}^{\;\mathrm{rk}}-{\vec{s}}_{3}^{\;\mathrm{ra}}∥}_{2}}\right)$$

(20c) $$R_{5}^{5^{\prime }}=f_{LCS}\left({\vec{r}}_{5},\frac{\left({\vec{s}}_{5}^{\;\mathrm{lh}}-{\vec{s}}_{5}^{\;\mathrm{lk}}\right)}{{∥{\vec{s}}_{5}^{\;\mathrm{lh}}-{\vec{s}}_{5}^{\;\mathrm{lk}}∥}_{2}}\right)$$

(20d) $$R_{6}^{6^{\prime }}=f_{LCS}\left({\vec{r}}_{6},\frac{\left({\vec{s}}_{6}^{\;\mathrm{lk}}-{\vec{s}}_{6}^{\;\mathrm{la}}\right)}{{∥{\vec{s}}_{6}^{\;\mathrm{lk}}-{\vec{s}}_{6}^{\;\mathrm{la}}∥}_{2}}\right).$$

The knee’s three rotation angles are then reported according to Grood and Suntay [82].

### 3.5. Selection of Noise Parameters

One major advantage of casting the estimation problem of Equation (18) only in terms of IMU pose trajectories (i.e., the minimal parameterization of the problem), rather than estimating the time-series pose trajectories of the underlying anatomical frames (c.f. [41,58]) is that model noise terms may be measured empirically. The IMUs in this experiment had a comparison reference—the 3-marker planes on which the IMUs were mounted. Additionally, markers were placed on the medial and lateral rotation points of the knee and ankle to compute the mocap-derived joint centers. The mocap hip center was derived from subject pelvic anthropometry according to Seidel et al. [96], using markers on the anterior and posterior superior iliac spine (ASIS and PSIS, respectively). From these joint center locations according to optical motion capture data, the mocap-estimated distance between the IMU pose and a neighboring joint center can be computed. For a single subject (S1), for each IMU and neighboring joint center combination, the empirical distribution of $e_{k}$ in Equation (10) and best-fit Gaussian covariance $\Sigma_{j}$ were found. This covariance was used for each subject afterward. Similarly for the knee hinge constraint, the empirical distribution of $e_{i}$ in Equation (8) for S1 was found and best-fit Gaussian covariance $\Sigma_{k}$ was used for each subject in the experiment.

### 3.6. Selection of Anthropometric Priors

The parameters of the anthropometric model (Section 2.2.5) were defined for the three types of anthropometric constraints present in this model (Table 2): (a) Distance between ankle and knee rotation centers for both legs, (b) distance between knee and hip rotation centers for both legs, and (c) distance between the two hip rotation centers. Note that as constraint (a) and (b) exist for both legs, there are five anthropometric constraints implemented in the model. If the anthropometric length $\mu_{L}$ is measured precisely for a given subject, associated variance $\sigma_{L}^{2}$ may be set to a low value to enforce a hard constraint on anthropometric length. However, in this study, and in many other applications, measuring multiple $\mu_{L}$ per subject is impractical. Therefore, these distributions were inferred from the literature.

The sources used for anthropometric measurements [97,98] report distributions separately for male and female subjects. As this study incorporated both male and female subjects, a composite distribution must be constructed. Given a measurement mean μ, standard deviation σ for each gender, the larger distribution of measurements was taken as $N(\left(\mu_{M}+\mu_{F}\right)/2,\sigma_{M}^{2}+\sigma_{F}^{2})$.

Additionally, the maximum/minimum anthropometric length constraint of Section 2.2.6 was implemented in this model. For the femur and tibia length, the minimum and maximum constraints were set as the 1st percentile female length and the 99th percentile male length, respectively. For femoral head separation, the maximum was set as the 99th percentile male hip breadth. However, as no suitable minimum could be found, this value was set to zero.

### 3.7. Other Priors

This current implementation has no absolute reference of position (e.g., landmarks or GPS) or absolute reference of heading (e.g., landmarks or magnetometers). This leads to a gauge freedom [89] of absolute position, velocity, and heading of the estimated solution. There are multiple approaches [99] to remedy gauge freedom and here, the simplest approach was taken. At the first keyframe for the lumbar IMU, a prior of zero position, zero velocity, and zero heading angle were set. This approach yielded an estimated solution which is accurate up to a constant offset of absolute position, velocity, and heading.

### 3.8. Initialization

For general nonlinear, nonconvex optimization, a consideration of the initial point is important. A good initialization of the method not only yields faster convergence, but also reduces the chance of convergence to local minima [100], which will typically produce nonsensical solutions. For this work, position, velocity, and IMU biases were initialized to zero, and orientations were initialized to the identity rotation matrix. Static vectors from IMUs to neighboring joint centers and knee axes were initialized from an assumed nominal alignment.

### 3.9. Hinge Axis Direction Disambiguation

As discussed in Section 2.2.9, one structural nonidentifiability of this problem is that both the positive and negative signed knee axes sign are equivalent solutions to Equation (8). This condition does not affect the convergence of the optimization problem however it will impact the ISB-consistent anatomical coordinate system definition that assumes knee axes point to the subject’s right (Section 3.4). The following steps were performed post-hoc (after the optimization and prior to joint angle derivation) to ensure that all axes point to the subject’s right:

(Step #1) Ensuring both knee axes are pointed in the same direction. First, the sign of ${\vec{r}}_{B}$ is adjusted to ensure it points to the same side as ${\vec{r}}_{A}$. Both knee axes are transformed into the global frame for all points in time through estimated IMU orientations $R_{A}^{N}$ and $R_{B}^{N}$. For each point in time, the angle between the knee axes in the world frame is computed. If the median of this distribution is greater than 90 degrees, we conclude that the knee axes point in opposite directions, and the sign of ${\vec{r}}_{B}$ is flipped. Otherwise, we conclude that knee axes are pointed in the same direction;
(Step #2) Ensuring both axes are pointed to the subject’s right. After Step #1, both knee axes will point to either the subject’s left or the subject’s right. However, if both axes point to the subject’s left, then the knee flexion/extension angle as derived in Section 3.4 will have the incorrect sign. Per the ISB-recommended knee angle convention (if both knee axes point to the subject’s right), the range of motion (ROM) of the knee angle should fall approximately in [+10${}^{\circ }$,−150${}^{\circ }$]. If both knee axes point to the subject’s left, this ROM will fall in [+150${}^{\circ }$,−10${}^{\circ }$]. Therefore, after Step #1 the knee angle is computed. If the median knee angle is greater than +20${}^{\circ }$, it is concluded that both knee axes must have been pointing to the subject’s left. Then both axes’ signs are flipped and the angle is recomputed.

### 3.10. Statistical Analysis

Hypothesis 1 considers the RMSE of estimated IMU pitch and roll for each IMU in the proposed model compared with a control method: The individual optimization-based smoothing of each IMU’s pose trajectory (i.e., without the biomechanical constraints of Section 2.2.2, Section 2.2.3, Section 2.2.4, Section 2.2.5, Section 2.2.6 and Section 2.2.7). For 12 subjects with 7 IMUs each, this produced 84 pitch and 84 roll RMSE values for each method. The 336 total RMSE values were considered as dependent measures of an analysis of variance (ANOVA) model. Categorical fixed effects were modeled for IMU, degree of freedom (i.e., pitch vs. roll), and model type (i.e., proposed vs. control). Subject was also included as a random effect. As each IMU can have a different accuracy in pitch or roll depending on the model used, an interaction term was also included. As the ANOVA fit for raw pitch/roll RMSE values was found to have right-skewed residuals, the model was refit using the $\log_{10}$ transform.

To characterize IMU position estimation accuracy of the proposed method, RMSE of the norm distance between adjacent IMUs is reported and computed for each subject as:

(21) $$\mathrm{RMSE}({∥p_{k,IMU}^{A}-p_{k,IMU}^{B}∥}_{2}-{∥p_{k,OMC}^{A}-p_{k,OMC}^{B}∥}_{2})\;\forall \;k=1\ldots M$$

where $p_{IMU}$ is the estimation position of the IMU, $p_{OMC}$ is the position of the IMU according to the 3-marker mocap clusters on each IMU, and (A,B)∈S is every adjacent pair of IMUs in the model. Note that the norm difference in position is reported rather than vector components because the proposed model has no guarantee of absolute heading reference, so it was intractable to align the world frame of the proposed model with the world frame of the optical motion capture system.

The motion capture based OpenSim-estimated joint angles (hereafter referred to as mocap angles) and the joint angles derived according to the proposed method (hereafter referred to as IMU angles) for the knee flexion/extension angle were compared. Both the RMSE and peak error of the difference between the estimated mocap angles and IMU angles are reported. While the term RMSE is used, it is noted that both signals are observations and contain sources of error.

## 4. Results and Discussion

The average length of the motion dataset was 89.57 s. The median wall time to run the optimization to convergence of motion dataset for each subject was 792.49 s. Tabulated results of pitch and roll RMSE for both the proposed and control methods are presented in Table 3 and Table 4, respectively. The ANOVA model of these pitch and roll RMSE values is presented in Table 5.

As an interaction effect of model type with IMU was found, the paired difference in RMSE between methods was considered. The [25th, 75th] percentile values inclusive of all IMUs was −0.49${}^{\circ }$ to +0.21${}^{\circ }$. Thus, while a significant difference was observed, these differences are not operationally relevant. One notable outlier was for roll error of the left shank IMU, where the paired difference in the RMSE value (control method − proposed method) was +1.59${}^{\circ }$, indicating that for this specific IMU the proposed method yielded a much lower roll error. It is concluded that hypothesis 1 is supported: The proposed method does not yield an operationally significant increase in error of IMU pitch/roll estimation. Note that yaw estimates were not analyzed, as the proposed method did not include any heading reference sensor. A motivation for this work was to estimate skeletal kinematics in a way which did not require an accurate absolute yaw estimation.

The RMSE of norm distance between adjacent IMUs are reported for each subject in Table 6. Average norm distance RMSE across all joints and subjects is 4.16 cm. These results support that the method robustly constrains the positional relationship between IMUs on the human body. The error is larger for the IMU pairs flanking the hip joints, i.e., the lumbar-thigh IMU pairs (8.16 cm mean RMSE for hip joints vs. 2.16 cm mean RMSE for all other joints). This may be due to significant thigh tissue motion through the 3DOF hip articulation. While these results demonstrate a constrained positional relationship between IMUs, future work is required to specify operationally-relevant position accuracy criteria for IMU-based human kinematics systems.

RMSE and peak error of IMU angles vs. mocap angles is presented in Table 7. Peak error represents the maximum absolute error observed over the entire trial for each subject. These results offer a few key takeaways. The proposed method adequately estimates knee flexion/extension without the use of magnetometers to provide IMU heading. Average knee flexion/extension angle RMSE was found to be 4.34${}^{\circ }$ across all subjects and both knees. These results are generally in line with the accuracy reported elsewhere in the literature, such as McGrath et al. [54]. However, McGrath et al. [54] used simulated IMU data and assumed the proximal IMU vector for the thigh and shank IMU, whereas this method estimates these quantities and uses real IMU data. Average peak error across all subjects and both knees was found to be 11.47${}^{\circ }$. The magnitude of peak error that is considered acceptable will depend on the operational scenario. Some joints may necessitate different requirements. Future work should consider what threshold of peak error is acceptable for specific applications.

Proposed model error may be partly explained by an imperfect estimation of the static vectors from the IMUs to the joint centers. If these joint centers are improperly estimated to lie slightly anterior or posterior of their true values, this would induce a static offset in the derived joint angles. Additional sources of error may come from imperfect mocap data, such as a mismatch between the placement of the physical mocap markers on the human subject and the virtual markers of the scaled OpenSim skeletal model. The OpenSim model is also imperfect, making assumptions about subject geometry and kinematic joint definitions. An error of all surface-mounted IMU-based methods is generally increased by soft tissue artifacts, i.e., perturbations of the IMU due to soft skin, fat, and muscle tissues which compress and deviate during human motion. Finally, knee angles are estimated without a heading reference. It is reasonable to believe that if the subject were in an environment that allowed for robust magnetometer usage (such as outdoors) and a well-calibrated magnetometer was available on the IMU package, that the estimated joint angles would have increased accuracy, because full orientation identifiability would be established rather than relying solely on the kinematics of the system (e.g., the hinge model) to provide information on the heading relationship between IMUs.

It should be noted that only the flexion/extension angle is compared for the knee, as the mocap angles are based on the OpenSim gait 2392 model, which is a 1DOF knee model. However, the proposed method also derives knee internal/external and adduction/abduction angles. Across all subjects, the [25th, 75th] percentiles of knee ROM were [33.2${}^{\circ }$, 44.2${}^{\circ }$] for internal/external rotation and [13.3${}^{\circ }$, 19.8${}^{\circ }$] for abduction/adduction. In comparison, Deesloovere et al. [101] reports a knee internal/external rotation ROM of 14.41${}^{\circ }$± 4.1${}^{\circ }$ during walking. Lafortune et al. [102] reports a knee abduction/adduction ROM of 10${}^{\circ }$ during the swing phase of gait however, no inter-subject variation of the five subjects studied is reported. Furthermore, Lafortune et al. attached the segment marker references to intracortical pins. This may reduce ROM compared to an IMU or mocap system which is confounded by soft tissue effects. The larger knee internal/external rotation and abduction/adduction ROM found in the current study are possibly due to (1) a motion task that differs from natural gait, (2) the variability from the selected sample, (3) suboptimal solutions from numerical optimization, or (4) noises within the system (e.g. soft tissue artifacts). As the knee articulates, the motion of muscle and underlying tissue alters the relative orientation between the IMU and anatomical coordinate frames, which may yield a larger ROM for internal/external and abduction/adduction.

### Future Work and Limitations

The current work uses anthropometric measures derived from standard populations. However, in many relevant use cases of this system, an assumption of standard anthropometry may not be appropriate (e.g., for gait analysis, where limb length discrepancies may drive abnormal gait). In these instances, the end user should specifically measure the anthropometry of the subject where possible to avoid increased error due to inappropriate anthropometric constraints in the model. Furthermore, the proposed method requires seven IMUs mounted on the lower body, which may not be tractable in certain settings.

Future extensions of the current work should also implement more robust initialization schemes that initialize the solution within the "basin of attraction" of the global minimum. For this work, a few initialization approaches were implemented, but none were shown to be more robust than the trivial “zero” initialization approach. This trivial initialization yields a limitation, as some poor results were almost certainly the result of emergent local minimum in which the optimization method became trapped. Additionally, other solution methodologies such as a simulated annealing [103] type approach may more robustly avoid local minima. Furthermore, IMU-based methods may be confounded by sensor bias. In the current work, time-series IMU gyroscope and accelerometer bias were estimated under a random-walk model with calibration parameters provided by the manufacturer. In the case that a high error is observed, it may be necessary to re-calibrate the sensor to update its calibration parameters and/or collect robust prior measurements of sensor bias. Other augmented approaches may be used to assess the quality of joint motion in order to reduce error. Ledel et al. [104] proposed a method based on an artificial neural network to classify the quality of a joint motion. It may be possible to perform the proposed estimation methodology only on data, which is assessed to be of high quality. If the application allowed, it is also possible to use external systems (e.g., camera systems [105]) to improve accuracy of IMU estimates.

The ease of use of the factor graph-based approach presented here allows for many possible extensions to this work to be neatly included as probabilistic constraints. These possible extensions include foot ground contact estimation [59] (while leveraging zero velocity updates and IMU position anchoring), the IMU-to-segment assignment problem [106], gait parameter estimation, activity/task identification, detection of IMU discrete shifting on the body, and many more.

The current work was validated against a scripted human motion profile task. While many functional calibration methods are present in the literature, a systematic study of these methods and which minimal set of motions sufficiently excite the degrees of freedom of the human skeleton may unify the numerous approaches. A robotic reference (rather than a human) may be useful in this pursuit—a robot may quickly reproduce motions while removing soft tissue noise. Moreover, the current considered task was chosen to excite all degrees of freedom of the skeleton. In more operationally-relevant tasks, e.g., gait, all degrees of freedom may not be excited, and further model assumptions or heuristic approaches may be utilized to aid model observability.

The vectors constraining the IMUs to their neighboring joint centers and joint rotation axes were assumed to be static in the proposed method. This assumption is not strictly true, due to soft tissue perturbation and the imperfect hinge kinematics of joints. Future work could consider a time-series bias model of these parameters to better capture these dynamics or perform further investigation to characterize these noise models.

The accuracy of knee internal/external rotation and abduction/adduction were unable to be directly assessed because the mocap reference model did not permit these DOF. As a result, only the ROM of these DOF were able to be compared to the literature. Future work should consider comparing against 3DOF models of the knee where appropriate, in order to better capture the effects of system noise on these DOFs. Additionally, all noise models in this work are assumed Gaussian and isotropic. Future work could also consider alternate noise modeling approaches or parameter estimation techniques [107] where appropriate. The proposed method can also be expanded to derive hip and ankle angles.

## 5. Conclusions

A method was developed for an optimization-based human lower body kinematics estimator without the use of magnetometers, requiring no functional calibration under human kinematics that sufficiently excite the lower body degrees of freedom. The proposed method adequately estimated knee flexion/extension to 4.34${}^{\circ }$ RMSE. This method directly estimated the knee’s rotation axis and positional relationship between IMUs and their adjacent joint centers, which enabled knee joints to be derived and reported using ISB convention. All results were presented for a scripted human motion profile task across 12 subjects. The method was shown to maintain the accuracy of IMU pitch/roll estimation once the biomechanical model was added. Relative norm positional RMSE between adjacent IMUs was 4.16 cm on average, supporting that the proposed method does robustly constrain IMU positional relationships. The method formulates a number of novel kinematic and anthropometric models of the human lower body, including a hinge model for the knee joint, inclusion of subject anthropometry from population data, femur/tibia segment length discrepancy, and modeling of the angle between the knee’s rotation axis and segment proximal direction. These novel constraints provide additional observability to the lower body kinematic model. This work was formulated as a factor graph and implemented in a framework which leveraged state-of-the-art IMU estimation techniques. The developed framework allows for expressive inclusion of new kinematics models, intended to provide a foundation for future work to build upon.

## Figures and Tables

**Table 1:** Distribution of angle between the knee’s rotation axis and the leg segments according to Hollister et al. [84] for use in Equation (12), adapted for a right-pointing knee axis and proximal-pointing segment definition.

Segment	μ	$\sigma_{o}$
Left femur	96${}^{\circ }$	2.4${}^{\circ }$
Left tibia	88${}^{\circ }$	1.2${}^{\circ }$
Right femur	84${}^{\circ }$	2.4${}^{\circ }$
Right tibia	92${}^{\circ }$	1.2${}^{\circ }$

**Table 2:** Assumed mean and variance for anthropometric priors in the proposed model. All values in meters.

Constraint	$\mu_{L}$	$\sigma_{L}$	$L_{\min}$	$L_{\max}$	Source
Tibial length	0.411	0.026	0.344	0.479	ANSUR II [97], *Calf Link*
Femur length	0.394	0.030	0.326	0.480	ANSUR II [97], *Thigh Link*
Femoral head separation	0.187	0.009	0	0.409	Rabari et al. [98], ANSUR II [97], *Hip Breadth*

**Table 3:** Pitch root mean square error (RMSE) (degrees) by subject and IMU for the proposed (P) method and the control (C) method.

	Sacrum		RThigh		RShank		RFoot		LThigh		LShank		LFoot	
Subject	P	C	P	C	P	C	P	C	P	C	P	C	P	C
**1**	0.96	1.31	1.49	1.46	1.58	1.51	2.05	3.28	1.31	0.60	1.13	1.73	1.90	2.03
**2**	1.08	1.20	1.56	2.12	0.95	1.65	1.72	2.67	1.38	1.63	0.97	2.26	1.23	1.82
**3**	2.56	1.12	2.60	2.00	1.81	1.20	2.50	2.22	1.43	2.12	1.95	3.43	2.09	1.91
**4**	1.42	2.07	1.20	0.60	1.71	1.67	1.06	4.34	0.92	0.83	0.84	1.27	2.11	1.58
**5**	1.33	1.64	1.05	1.02	1.15	1.01	1.08	3.45	0.99	0.94	0.88	1.01	2.17	2.11
**6**	0.24	0.16	0.72	0.88	1.60	1.41	1.81	1.60	0.66	0.55	0.46	0.74	0.67	0.86
**7**	2.02	0.39	2.12	1.22	2.74	1.74	1.90	2.46	2.11	0.96	1.98	0.84	1.94	0.75
**8**	2.03	0.28	1.75	1.16	1.72	1.71	3.48	1.49	2.50	0.28	2.23	1.04	2.01	0.89
**9**	0.87	0.52	1.30	1.35	2.25	2.19	1.16	1.69	0.43	0.59	0.37	0.74	0.61	0.61
**10**	0.68	0.80	0.45	0.65	1.55	1.29	1.41	1.55	0.59	1.31	0.55	1.20	0.75	1.35
**11**	0.39	0.29	0.77	0.60	1.21	1.28	2.16	1.86	0.59	0.55	0.51	0.58	0.59	1.18
**12**	3.98	1.87	4.38	0.42	3.26	1.22	5.26	1.48	4.70	0.58	4.19	0.63	4.24	1.05
**Mean**	1.46	0.97	1.62	1.12	1.79	1.49	2.13	2.34	1.47	0.91	1.34	1.29	1.69	1.35

**Table 4:** Roll RMSE (degrees) by subject and IMU for the proposed (P) method and the control (C) method.

	Sacrum		RThigh		RShank		RFoot		LThigh		LShank		LFoot	
Subject	P	C	P	C	P	C	P	C	P	C	P	C	P	C
**1**	3.45	0.59	2.92	2.19	3.19	1.47	2.79	2.43	2.42	1.62	2.60	1.43	2.92	1.05
**2**	1.17	1.02	0.40	4.70	2.07	2.30	0.60	1.14	0.70	0.71	0.43	3.90	0.38	0.96
**3**	4.15	0.57	3.24	3.48	4.14	1.69	4.12	1.40	3.09	0.90	3.66	5.07	3.74	2.93
**4**	2.52	1.17	2.92	2.03	3.49	1.70	2.68	1.49	2.44	1.04	2.63	4.42	2.48	0.86
**5**	2.36	1.32	0.76	0.74	1.94	1.67	0.93	0.77	1.03	1.38	1.22	1.60	1.06	0.79
**6**	0.50	0.88	0.75	1.77	2.07	2.06	1.87	1.86	0.56	1.76	1.34	3.36	0.96	3.49
**7**	0.51	1.00	1.26	2.31	2.61	2.45	1.24	2.02	1.26	1.89	1.25	1.06	0.68	1.66
**8**	0.78	0.77	1.03	2.06	1.52	1.54	1.21	1.67	0.75	1.36	0.67	3.98	0.54	2.15
**9**	2.72	0.92	2.35	3.50	3.19	1.78	2.66	1.56	2.38	1.25	1.74	4.94	2.12	2.11
**10**	0.81	0.81	1.65	2.64	1.43	2.03	1.05	1.71	1.60	2.31	1.02	3.03	0.83	2.38
**11**	0.99	1.23	1.43	1.03	1.72	2.26	1.44	1.88	1.17	1.90	0.79	1.30	0.93	5.16
**12**	0.51	1.13	1.44	1.20	2.03	1.14	1.55	1.51	0.67	1.89	1.36	3.77	0.83	1.58
**Mean**	1.71	0.95	1.68	2.30	2.45	1.84	1.84	1.62	1.51	1.50	1.56	3.16	1.46	2.09

**Table 5:** ANOVA model of estimated IMU pitch/roll RMSE.

Source	Sum Sq.	d.f.	Mean Sq.	F	*p*
Subject	3.04	11	0.28	5.09	<0.001
IMU	2.56	6	0.43	7.86	<0.001
Model	2.0 × 10${}^{-3}$	1	2.0 × 10${}^{-3}$	0.04	0.85
DOF	0.79	1	0.79	14.58	<0.001
IMU*Model	0.92	6	0.15	2.82	0.01
Error	16.83	310	0.05		
Total	24.14	335			

DOF refers to IMU degree of freedom, i.e., pitch and roll.

**Table 6:** RMSE in centimeters of total distance between each IMU pair, per subject.

Subject	Lumbar RThigh	RThigh RShank	RShank RFoot	Lumbar LThigh	LThigh LShank	LShank LFoot
**1**	16.92	0.92	3.21	8.79	4.35	2.23
**2**	8.28	0.90	2.33	7.30	0.66	2.74
**3**	4.86	2.79	3.20	5.27	2.84	3.67
**4**	5.93	1.84	2.30	6.04	1.83	2.65
**5**	13.53	1.27	2.72	1.36	2.68	3.34
**6**	4.86	2.29	2.87	5.25	3.38	3.79
**7**	9.90	0.73	2.38	8.58	0.57	2.68
**8**	11.44	0.49	2.52	10.73	1.46	1.90
**9**	13.65	0.92	2.45	10.99	0.68	3.07
**10**	8.51	1.17	1.94	4.72	1.96	2.61
**11**	10.19	0.97	2.40	6.11	1.53	3.12
**12**	5.75	0.47	2.42	6.81	1.89	2.49
**Mean**	9.49	1.23	2.56	6.83	1.98	2.86
**Std**	3.71	0.69	0.36	2.58	1.11	0.54

**Table 7:** Error (degrees) of IMU joint angles vs. mocap joint angles for the motion profile dataset. *F/E* refers to flexion/extension of the joint.

	RMSE		Peak Error	
Subject	RKnee *F/E*	LKnee *F/E*	RKnee *F/E*	LKnee *F/E*
**1**	2.17	2.08	8.83	6.00
**2**	3.28	7.09	8.70	10.85
**3**	5.37	10.30	21.84	15.42
**4**	2.37	3.34	8.45	9.13
**5**	4.61	6.74	10.66	11.37
**6**	4.28	4.33	18.69	10.41
**7**	3.32	5.60	9.75	11.85
**8**	3.11	4.92	15.45	13.94
**9**	3.83	7.71	14.45	12.51
**10**	2.95	3.97	10.67	7.85
**11**	4.22	2.31	11.24	9.46
**12**	2.19	4.10	9.46	8.30
**Mean**	3.47	5.21	12.35	10.59
**Std**	1.01	2.40	4.34	2.66
